# Prospects for the Use of Cannabinoids in Psychiatric Disorders

**DOI:** 10.3389/fpsyt.2021.620073

**Published:** 2021-03-12

**Authors:** Michał Graczyk, Małgorzata Łukowicz, Tomasz Dzierzanowski

**Affiliations:** ^1^Department of Palliative Care, Collegium Medicum in Bydgoszcz, Nicolaus Copernicus University in Toruń, Toruń, Poland; ^2^Department of Rehabilitation, Center of Postgraduate Medical Education, Gruca Orthopedic and Trauma Teaching Hospital in Otwock, Otwock, Poland; ^3^Laboratory of Palliative Medicine, Department of Social Medicine and Public Health, Medical University of Warsaw, Warsaw, Poland

**Keywords:** cannabis, cannabinoids, psychiatric disorders, anxiety, depression, dementia, sleep disorders, substitution therapy

## Abstract

Increasing evidence suggests an essential role of the endocannabinoid system in modulating cognitive abilities, mood, stress, and sleep. The psychoactive effects of cannabis are described as euphoric, calming, anxiolytic, and sleep-inducing and positively affect the mood, but can also adversely affect therapy. The responses to cannabinoid medications depend on the patient's endocannabinoid system activity, the proportion of phytocannabinoids, the terpenoid composition, and the dose used. There is some evidence for a therapeutic use of phytocannabinoids in psychiatric conditions. THC and CBD may have opposing effects on anxiety. Current guidelines recommend caution in using THC in patients with anxiety or mood disorders. In a small number of clinical trials, cannabinoids used to treat cancer, HIV, multiple sclerosis, hepatitis C, Crohn's disease, and chronic neuropathic pain report decreases in anxiety or depression symptoms and presented sedative and anxiolytic effects. Several studies have investigated the influence of potential genetic factors on psychosis and schizophrenia development after cannabis use. THC may increase the risk of psychosis, especially in young patients with an immature central nervous system. There is limited evidence from clinical trials that cannabinoids are effective therapy for sleep disorders associated with concomitant conditions. There is evidence for a possible role of cannabis as a substitute for alcohol and drugs, also in the context of the risks of opioid use (e.g., opioid-related mortality). In this narrative review of the recent evidence, we discuss the prospects of using the psychoactive effects of cannabinoids in treating mental and psychiatric disorders. However, this evidence is weak for some clinical conditions and well-designed randomized controlled trials are currently lacking. Furthermore, some disorders may be worsened by cannabis use.

## Neuromodulating Role of the Endocannabinoid System

Cannabis affects the nervous system in four main domains:

1. Mood (euphoria, unfounded laughter; paranoid or anxious reactions at high doses),
2. Perception (disturbance of the perception of time and space),
3. Somatic symptoms (fatigue, problems with motor coordination, dizziness),
4. Cognitive impairment (confusion, impaired concentration, impaired short-term, and working memory) (1).

Over 110 cannabinoid receptors' ligands have been isolated from *Cannabis sativa*, of which some have neuromodulating properties (2). In the Nineteenth and twentieth centuries, hemp was used to treat sleep disorders, pain, and increase appetite (3). Since the 1990s, after the discovery of the endocannabinoid system (ECS) (4), many publications explaining the mechanism of its action appeared. CB_1_ receptors can be found mainly in the central and peripheral nervous systems (5, 6). When discussing the effects of cannabis on the CNS, one should distinguish the effects of the two main cannabinoids, D9-tetrahydrocannabinol (THC) and cannabidiol (CBD), with only THC and its metabolites having a psychoactive effect (2).

Increasing evidence suggests an essential role of the endocannabinoid system (ECS) in the regulation of cognitive abilities, mood, stress, and sleep (7–9). In animal models, pharmacological or genetic disruption of endocannabinoid signaling results in a neurobehavioral response that imitates the classic stress response. It is manifested by activation of the hypothalamic-pituitary-adrenal axis (HPA), increased anxiety, excessive vigilance, agitation, inhibition of feeding behavior, decreased response to rewarding stimuli, and impaired cognitive flexibility (9). Regulation of the stress-response mechanism in short-term stress causes ECS inhibition, while long-term stress stimulates ECS, which alleviates the negative effects of the stressful situation (8). Endocannabinoids (via CB_1_ receptors) modulate the functions of all hypothalamic-pituitary-gland axes. Chronic stress seems to reduce the ECS's ability to buffer stress and may induce psychopathology, including anxiety and depression (8). The ECS signaling modulates HPA axis activity in stressful conditions, which may promote psychiatric disorders (10).

The CB_1_ receptor plays an inhibitory role in the release of excitatory amino acids and GABA which consequently regulate the release of other transmitters: acetylcholine, dopamine, histamine, serotonin, noradrenaline, prostanoids, and opioid peptides (11, 12). CB_1_ receptors are present at very high levels on inhibitory (GABAergic) interneurons and at a lesser extent on excitatory (glutamatergic) terminals (13), as well as on neurons expressing dopamine D_1_ receptors, playing a specific role in the repertoire of different emotional behaviors, including social and cognitive activity, which are affected in psychiatric disorders (14–17).

By activating the CB_1_ cannabinoid receptor, THC may induce euphoria, cognitive impairment, and intensify negative emotional states, including anxiety. In cell research, CBD has been shown to reduce the CB_1_ cannabinoid receptor's activation but is not an antagonist, as it induces intracellular sequelae. CBD may function as a negative allosteric modulator (NAM) of the CB_1_ receptor and binds to it at a completely different site than the target THC binding site (18). In the biochemical studies, cannabidiol enhanced endogenous anandamide signaling indirectly through inhibition of the intracellular degradation of anandamide catalyzed by the enzyme fatty acid amide hydrolase (FAAH) (19, 20). The inhibition of FAAH may not be relevant for humans but CBD may inhibit the transport of anandamide (AEA), oleoylethanolamide (OEA) and palmitoylethanolamide (PEA) to FAAH by blocking fatty acid-binding proteins (FABPs), which are intracellular carriers for Δ9-tetrahydrocannabinol (THC) and cannabidiol (CBD) (21).

The recent findings suggest that possibly relevant mechanisms of CBD encompass the facilitation of serotonergic neurotransmission via allosteric 5-HT_1_A receptor modulation, modulation of glucose homeostasis and inflammatory processes by PPARγ activation, and the interaction with the transient receptor potential vanilloid-1 receptor (TRPV1) (22, 23). Recent evidence suggests the therapeutic potential of cannabinoid-based drugs for a wide range of medical conditions, including neurological and psychiatric disorders (24).

## Possible Clinical Indications of Cannabis and Its Derivatives

### Mood Disorders

The psychoactive effects of cannabis are described as euphoric, calming, anxiolytic, and sleep-inducing. Some of them positively affect mood. On the other hand, in some persons, adverse effects, such as paranoia, irritation, dysphoria, depression, depersonalization, and demotivation, appear (25, 26) (Supplementary Table 1). The reactions may depend on the patient's ECS activity, the proportion of phytocannabinoids, the terpenoid composition, and the dose used (bell effect, stimulating effect at a low dose, and inhibitory effect at a high dose) (27). The interaction between these effects can be complex, and therefore requires selecting the appropriate variety and dose by an experienced professional. Noteworthy, the balance of positive and negative effects can change in the same patient during observation and treatment (27). A patient experiencing a mood disorder may not be objective in assessing his or her condition and cannot decide on his or her own to modify treatment. Therefore, professional care and control are essential.

The mood-elevating properties of cannabinoids have been known for a long time and are considered non-toxic. Many patients who do not respond appropriately to standard pharmacological treatments of depression may benefit from medical cannabis use. Cannabinoids may have therapeutic potential in both depression and bipolar disorder (28). The duality of bipolar disorder makes it challenging to treat. Standard pharmacotherapy does not always help with all symptoms and stabilizes both manic and depressive episodes. Some patients successfully add cannabis to ongoing pharmaceuticals, enhancing their effects, or reducing the side effects of therapy (28, 29). There are reports that cannabis can be a mood stabilizer in bipolar disorder and an adjuvant to lithium therapy (it allows for dose reduction) (28). Nevertheless, there is limited clinical evidence suggesting that cannabis use may cause the onset and worsen the clinical course in bipolar disorder (30, 31).

Early reports in Western medicine describe its effects as “mental joy.” Today, many patients admit to using marijuana to relieve symptoms of depression. In an Australian study, 56% of medical cannabinoid users surveyed used them for depression (32).

In U.S. states which legalized marijuana, suicide rates among men aged 20–39 have decreased compared to states where marijuana is illegal (33).

Often depression is secondary to a life-limiting illness. Clinical observations indicate that cannabinoids may provide new treatment options for anxiety or depression secondary to certain chronic diseases. In a study in HIV-infected patients, 86% reported an improvement in depression and 93% in anxiety (34).

In some observational studies of limited quality, cannabis containing CBD and THC in equal proportions attenuated some mood disorders reported in patients using THC-predominant cannabis. An observational study was conducted on 100 patients who used cannabis for multiple sclerosis, chronic pain, nausea, cancer, or psychological problems (35). Patients who used cannabis with low concentrations of cannabinoids (6% THC and 7.5% CBD) experienced significantly less anxiety, dejection, sadness, or depression. They also reported less appetite stimulation compared to those who reported using strains rich in THC (19% THC, <1% CBD) or with medium THC concentration (12% THC, <1% CBD (35). In another observational study, 75 patients suffering from depression, stress, and burnout syndrome were successfully treated with dronabinol, either alone or in combination with other antidepressants. Dronabinol appeared to be a successful antidepressant in a general medicine practice, alone or in combination with other antidepressants (36).

Evidence from a small number of clinical trials of prescribed THC-containing cannabis that patients using cannabinoids to treat cancer, HIV, MS, hepatitis C, Crohn's disease, or chronic neuropathic pain report relief in symptoms of anxiety or depression (9, 35, 37–39).

Most of the psychoactive effects of medical marijuana, such as euphoria, do not occur in every patient. Moreover, less frequently, anti-euphoric, or dysphoric reactions are also observed (40). Patients taking marijuana may experience different effects depending on their current mood, treatment expectations, drug mix, and dosages. If taken at the “wrong time” or during the decreased mood, it can provoke negative thoughts (27). It is essential in adolescents in whom there is a greater risk of depression, other mental disorders, and suicide later in life (41). This can be explained by the immaturity of the central nervous system and neural connections. Still, it is difficult to say whether cannabinoids caused depression or were used in response to depression.

Evidence from pre-clinical and clinical studies indicates a vital role for the ECS in anxiety and mood disorders. The decreased endocannabinoid signaling may entail a depressive-like phenotype. Therefore, boosting of endocannabinoid signaling may appear a novel therapeutic option for the treatment of depression (42). Low doses of CB_1_ receptor agonists reduced anxiety behavior and increased antidepressant-like responses in animals (43). Moreover, similar to typical antidepressants, CB_1_ receptor agonists seem to increase the central transmission of neurotransmitters (serotonin, noradrenaline) (44, 45). In support of this theory, rimonabant (CB_1_ receptor antagonist), approved to treat obesity, was withdrawn after reports of mood and sleep disorders in persons who used it. Patients became more irritable and agitated, and an increase in the incidence of depression and even suicide were noted (46, 47). Despite the psychiatric side effects of rimonabant, there is still interest in the development of CB_1_ antagonism as a pharmacological tool for the treatment of metabolic disorders, with a better safety profile. In this context the peripheral CB_1_ blockade seems to be a promising therapeutic target (48).

Thus, CB_1_ receptors are an important new target in the development of antidepressants. However, the challenge of discovering new cannabinoid antidepressants is to develop CB_1_ agonists with selective antidepressant properties, which would reduce adverse psychotropic effects of cannabis use to the smallest possible degree (44).

### Anxiety

Cannabis rich in THC induces anxiety behavior. The effect seems to be dose-dependent, with low doses having potentially anxiolytic properties and high doses being ineffective or even increasing anxiety levels (49). When taken in high doses by cannabis-naïve users, THC can cause intense fear and anxiety up to a panic attack. In contrast, long-term cannabis users report reduced anxiety, increased relaxation, and relief from tension (50).

CBD's anxiolytic effects have been assessed in animal models of generalized anxiety disorder, social phobia, panic disorder, obsessive-compulsive syndrome, and post-traumatic stress disorder (PTSD) and in humans (51–53). CBD use's positive anxiolytic effects have been observed in people with generalized social anxiety disorder (SAD) and effectively treat other anxiety disorders and reduce anxiety symptoms (54, 55). The results suggest that CBD reduces anxiety in patients with SAD and this is related to its influence on activity in limbic and paralimbic areas of the brain (56). The anxiolytic properties of CBD have been confirmed in humans and follows the same pattern of an inverted U-shaped dose-effect curve observed in many animal studies. It is necessary to determine the optimal therapeutic doses of CBD for introduction / implementation into clinical practice (57).

Cannabinoids have a sedative and anxiolytic effect and may be assessed by some patients as better than traditional drugs because they do not dull cognitive processes. Still, a significant proportion of patients have the opposite impression and report mental confusion after their use (27). It is commonly believed that a “laid-back” state is felt after cannabis use, but also anxiety can be exacerbated, even up to a panic attack (58). This problem is troublesome for inexperienced patients without prior training in using the cannabinoids, especially with high THC levels or too high an initial dose (59).

### Sleep Disorders

Cannabis and THC have a dose-dependent effect on sleep, with low doses to reduce sleep onset latency and increase slow-wave sleep and total sleep time; and high doses to cause sleep disturbances (60–63).

There is limited evidence from clinical trials that cannabis or THC improve sleep in patients with sleep disorders associated with comorbidities (obstructive sleep apnea syndrome, fibromyalgia, chronic pain, and multiple sclerosis) (64–66). Numerous studies on managing chronic pain, fibromyalgia, and multiple sclerosis report improved sleep in patients, though (67, 68).

Few reports suggest CBD improve REM sleep disturbances and excessive daytime sleepiness (55, 69).

### Schizophrenia and Psychosis

Significant evidence from epidemiological, pre-clinical, and clinical studies supports the association between THC (and THC-rich cannabis) and an increased risk of psychosis and schizophrenia (70, 71). However, it seems unlikely that they contribute to the development of mental illness (72). In contrast, based on a genetic approach, cannabis use was associated with an increased risk of schizophrenia than in non-users (73, 74). THC has a pro-psychotic effect, while CBD reduces the occurrence of such disorders (2, 19, 75). However, interactions between THC and CBD may be clinically significant (76).

THC might affect schizophrenia patients differentially causing transient exacerbation of psychotic and cognitive deficits in comparison to control subjects (77). THC-rich varieties may increase the risk of psychosis–especially in young patients whose brains are still developing (74). Novel cannabis strains contain far more THC than old strains, where for centuries, the ratio of THC to CBD has been comparable (2). The cannabis use by adolescents may change the endocannabinoid signaling and pose a potential environmental risk to develop psychosis. In pre-clinical and clinical studies, a potential role of the ECS both in pathophysiology of schizophrenia and as potential therapeutic target, has been found (78).

The evidence does not support or disapprove of CBD as an effective drug for schizophrenia or schizophrenic psychosis. However, emerging evidence suggests CBD's attenuating effect on THC-induced psychosis (19, 76, 79, 80). In a recently published randomized clinical trial of cannabidiol vs. placebo for cannabis use disorder, cannabidiol 400 and 800 mg doses appeared well-tolerated and effective at reducing cannabis use (81). In another randomized clinical trial, an antipsychotic effect of lower doses as an addo-on the multiple antipsychotics in chronically ill patients was not found. However, CBD appeared well-tolerated with no worsening of mood, suicidality, and movement side effects (82).

In several studies, the influence of potential genetic factors on psychosis and schizophrenia development, specifically the interaction function with cannabis use, have been investigated. In adolescence and early adulthood, exposure to various stimuli, including cannabis, can impair the ordinary course of neurobiological development and induce the early onset of schizophrenia in people with a genetic pre-disposition (83, 84). In human peripheral blood mononuclear cells (PBMCs) of schizophrenic subjects, selective alterations of DNA methylation at the promoter of the gene coding for the type-1 cannabinoid receptor (CNR1) were observed and confirmed in a well-validated animal model of schizophrenia, induced by prenatal methylazoxymethanol acetate (MAM). The degree of CNR1 DNA methylation in PBMCs may appear a potential biomarker for schizophrenia (85). In the neurodevelopmental MAM model of schizophrenia, a specific alteration of CB_1_ receptors in the pre-frontal cortex, fully reversed by cannabidiol treatment, was found, which may appear a novel potential antipsychotic (86). Likewise, a specific alteration of dopamine D_3_ receptors in the MAM model of schizophrenia was found, which seems to be a target of cannabidiol treatment (87). In the THC model of psychopathology there was a specific alteration of CB_1_ and D_2_ receptors in the pre-frontal cortex of rats, similarly to schizophrenic subjects, which was fully reversed by cannabidiol treatment, as novel potential antipsychotic (88). It is crucial that studies biologically quantify cannabinoid exposure, besides the self-reported use, when investigating its impact on cannabis-related mental health issues (i.e., psychosis, mood disorders, addiction) (89).

### Cognitive Disorders and Dementia

Cannabis is associated with cognitive impairment, including short-term memory, attention, executive functions, and psychomotor reaction, and this effect seems residual in heavy users (90, 91). On the other hand, in pre-clinical studies, the ECS demonstrated a protective effect against excitotoxicity, oxidative stress, and inflammation associated with the development of Alzheimer's disease (AD) (92). Animal studies have shown that ultralow doses of THC (0.002 mg/kg) slow down the formation of plaque and tangles and reduce the inflammation caused by their presence, thus supporting the treatment of dementia (93). In post-mortem brain tissue of AD patients and in experimental models of AD, a decrease in neuronal cannabinoid CB_1_ receptors, an increase in glial cannabinoid CB_2_ receptors, and over-expression of free acid amide hydrolase in astrocytes hint its potential role in inflammatory processes and in neuroprotection (94). Early pharmacological enhancement of brain endocannabinoid levels might protect against beta-amyloid neurotoxicity and its consequences (95). Moreover, beta-amyloid fragments induce a dose-dependent memory deficit, and this effect may be associated with cannabinoid CB_1_ receptors in the brain (96).

The clinical evidence for cannabinoids for the treatment of AD is scarce. In a Cochrane (2011) systematic review, the evidence did not support their effectiveness at improving disturbed behavior or other symptoms in dementia (97). However, only one small-size study met the inclusion criteria Volicer et al. (98). In a newer RCT, with 50 patients enrolled, low-dose THC did not significantly reduce dementia-related neuropsychiatric symptoms, though it was well-tolerated (99).

### Opioid Withdrawal Symptoms and Drug Substitution

The first report on the role of marijuana in treating substance abuse (including opiates) was published in The Lancet in 1889 (100).

There is growing evidence to support medical cannabis as an adjuvant or substitute for prescription opioids to treat chronic pain. Cannabinoids combined with opioid analgesics bring on hyper-additive pain relief, which results in a reduction in opioid use and opioid-induced adverse effects (101). Besides that, cannabinoids may prevent the development of opioid tolerance and withdrawal and may even resume the analgesic effect of opioids when the previous dose has become ineffective (101).

Studies show that the use of cannabinoids may be both safe and effective, also in elderly patients, and reduce the number of prescription drugs they receive, including opioids (25).

In a study conducted in Philadelphia, 91 opiate-addicted patients received methadone substitution therapy (102). Patients who used marijuana before the treatment needed less methadone. Additionally, using cannabis during methadone therapy resulted in less expressed withdrawal symptoms (assessed according to the Clinical Opiate Withdrawal Scale, COWS). The consumption of marijuana in the initial phase of substitution therapy, when strongly expressed withdrawal symptoms were present, was higher than in the subsequent ones when withdrawal symptoms retreated (102). Another randomized clinical trial revealed the potential of CBD to reduce cue-induced craving and anxiety as a treatment option for opioid use disorder (103).

The effectiveness of cannabinoids in alleviating withdrawal symptoms associated with opioid abstinence or dose reduction of opioid analgesics can be explained by overlapping neuroanatomical distribution, convergent neurochemical mechanisms, and comparable functional neurobiological properties of ECS and the opioid system (104, 105).

Medical cannabis's benefits were assessed in Canada on 404 patients in an anonymous survey that subjectively assessed the effects of medical cannabis on the use of alcohol and illicit psychoactive substances (106). Cannabinoids reduced withdrawal symptoms and resulted in less frequent side effects and better control of symptoms of existing diseases than other pharmaceuticals.

There is a growing consideration in substituting alcohol, opioids, and other psychoactive substances with cannabis to reduce abstinence-associated withdrawal symptoms and the risks of their use (e.g., opioid-related mortality). Pre-clinical studies suggest that some cannabinoids (such as THC) may ease opioid withdrawal (107, 108). In an observational study, cannabis use alleviated opioid withdrawal symptoms, but the clinical evidence is insufficient to draw any conclusive recommendation (109, 110).

Nevertheless, it is essential to reiterate that long-term cannabis use can cause a motivational syndrome and addiction (111).

## Conclusion

The neuroprotective role of the endocannabinoid system is still the subject of extensive research. Preclinical research suggests its modulatory effect on numerous neurological, emotional, and psychiatric symptoms. As discussed in this article, there is weak evidence for cannabinoids' beneficial results in anxiety, mood, and sleep disorders, as summarized in Table 1. There is also a growing interest in cannabis use as a substitute for psychoactive substances. On the other hand, several studies report the development of psychosis and cognitive impairment. The evidence supporting cannabis use in psychiatric disorders is insufficient and of low quality yet. Further translational research is necessary to understand the pharmacodynamics in humans, and clinical studies are required to assess the risks and benefits of cannabis use.

**Table 1:** The neuropsychiatric effects of tetrahydrocannabinol (THC) and cannabidiol (CBD) (2).

**THC**	**CBD**
• Psychoactive (euphoria or dysphoria, anxiety in some new users)	• No psychoactive activity • Counteracts psychotropic effects of THC (short-term memory and cognitive disorders) • Possible anti-psychotic effect
• Relaxation and bliss	
• Analgesic	• Analgesic
	• Anxiolytic and antidepressant
• Soporific (secondary effect, dose-dependent)	• Induces sleep, suppresses waking-up, regulates sleep disorders (also anxiety related)
• Stimulates appetite	• Suppresses appetite

## Author's Note

There is an increasing interest in cannabis use in neuropsychiatry. The evidence is relatively scarce or of low quality or comes from the pre-clinical research. Nevertheless, it supports the endocannabinoid system's role in regulating stress, mood, cognitive abilities, and sleep. The effects such as euphoric, anxiolytic, calming, or sleep-inducing are well known from recreational use experiences. There is increasing evidence for cannabinoids in the therapeutic use for anxiety, depression, insomnia, psychoses, and opioid substitution. In this mini-review, we tried to signal the research's key directions on cannabis in psychiatry. Where possible, we presented the clinical evidence to provide an overview of the state of knowledge. We avoided too detailed explanation of the pathophysiology or etiology, bearing in mind that the special issue “The Endocannabinoid System: Filling the Translational Gap between Neuroscience and Psychiatry” will consist of translational research articles and problem-specific papers. We hope that the article will be a kind of roadmap of the prospects of current and research.

## Author Contributions

MG: review plan. MG, MŁ, and TD: data collection, interpretation, and proof reading. MG and TD: writing. All authors contributed to the article and approved the submitted version.

## Conflict of Interest

The authors declare that the research was conducted in the absence of any commercial or financial relationships that could be construed as a potential conflict of interest.
