# Supplementary material for: Prospects for the Use of Cannabinoids in Psychiatric Disorders
Source: Front Psychiatry. 2021 Mar 12;12:620073. doi: 10.3389/fpsyt.2021.620073 (PMC7994770; doi:10.3389/fpsyt.2021.620073)
Supplement: Supplementary file 1 [file Table_1.pdf]

Table S1. The summary of clinical research

Study (year)	Study Design and Comparator (if applicable)	Number of Participants or Studies	Drug/Substance	Dosage mg/d	Condition	Treatment/use duration	Endpoints/Measures	Outcomes	Limitations
**Mood disorders**									
Abuhasira (2018) [25]	questionnaire-based telephone interview	901	THC reach strains and CBD reach strains of herbal cannabis	n/a	elderly patients (≥ 65 years)	6 months	• QoL (Likert scale)<br>• Pain intensity (NVS 0-10)<br>• Perception of the general effect of cannabis (Likert scale)<br>• Treatment success (defined as moderate or significant improvement in patient's condition and compliance)	Significant reduction in pan intensity (from NVS 8 to 4);<br>QoL improved; general effect of cannabis positive in 93.7% of the respondents (significant improvement in 41.9%) treatment success in 59.1% of the respondents	Telephone interviews; high bias; very poor quality of statistics.
Whiting (2015) [26]	systematic review and meta-analysis	6,462 participants in 79 trials	herbal cannabis	stratified per intervention and administration method	nausea and vomiting due to chemotherapy, appetite stimulation in HIV/AIDS, chronic pain, spasticity due to multiple sclerosis or paraplegia, depression, anxiety disorder, sleep disorder, psychosis, glaucoma, or Tourette syndrome	n/a	• symptom incidence and intensity	There was moderate-quality evidence to support the use of cannabinoids for the treatment of chronic pain and spasticity. There was low-quality evidence suggesting that cannabinoids were associated with improvements in nausea and vomiting due to chemotherapy, weight gain in HIV infection, sleep disorders, and Tourette syndrome. Cannabinoids were associated with an increased risk of short-term AEs (adverse events).	High-quality review. A number of methodological weaknesses was identified in the included trials including failure to appropriately handle withdrawals, selective outcome reporting, and inadequate description of methods of randomization, allocation concealment, blinding; very small sample size. (glaucoma, Tourette syndrome, sleep disorder, and anxiety disorder).
Moore (2007) [30]	systematic review and meta-analysis	35 studies	herbal cannabis	n/a	chronic pain (57%), depression (56%), arthritis (35%), persistent nausea (27%), and weight loss (26%)	n/a	• adjusted odds ratios and 95% CI for psychosis, depression, suicidal ideation, and anxiety	Increased risk of any psychotic outcome in individuals who had ever used cannabis, dependent on dose and frequency.<br>Results for depression, suicidal thoughts, and anxiety outcomes inconsistent, and fewer attempts were made to address non-causal explanations, than for psychosis.	--
Botsford (2020) [31]	systematic	47 studies; 203,012	marijuana	n/a	cannabis users	0.33-40 years	• the onset and course of depression, bipolar	Cannabis use is linked to the onset and poorer	Limited number of studies that met inclusion criteria,

	review of RCT and observational studies	patients analyzed in cohorts					• disorder, anxiety disorders (ADs), and post-traumatic stress disorder (PTSD)<br>• the therapeutic potential of cannabis and cannabinoids for these disorders	clinical course in bipolar disorder and PTSD, but this finding is not as clear in depression and ADs.	especially for longitudinal studies of cannabis on the clinical course of mood and Ads.<br>Few high-quality studies of cannabinoid pharmaceuticals in clinical settings available.
Swift (2005) [32]	questionnaire survey	147 responders	herbal cannabis	n/a	pain, nausea, and insomnia	n/a	• medical conditions/symptoms experienced,<br>• patterns of medical cannabis use,<br>• symptom relief and effects of use,<br>• comparison of cannabis to other medications, source and legal concerns (e.g., arrest),<br>• other concerns over use, opinion of family,<br>• friends and medical personnel, and<br>• interest in participating in a cannabis trial	"Great relief" overall in 86% respondents; substantial relief of specific symptoms such as pain, nausea and insomnia; nearly one half (41 %) experienced conditions or symptoms that were not helped by its use.	Concerns related to illegality.<br>High bias.
Anderson (2014) [33]	retrospective analysis of state-level suicide data	n/a	herbal cannabis	n/a	states with 'medical marijuana' legalized vs. control states	n/a	• suicides per 100,000 population	Suicide incidence among men aged 20 through 39 years decreased after medical marijuana legalization.	The association between legalizing medical marijuana and suicides was not statistically significant at the .05 level. Legalization was associated with a 10.8% and 9.4% reduction in the suicide rate of men aged 20 through 29 years and 30 through 39 years, respectively.
Woolridge (2005) [34]	cross-sectional questionnaire survey	523	non-medical source of supplied self-administered smoked products	n/a	pain and other medical symptoms in HIV patients	n/a	• cannabis use ration for symptom management<br>• Likert-style scale for lack of appetite, feeling sick (i.e., nausea), tremor, depression, anxiety, weight loss, weakness, tiredness, vision dimness, slurred speech, memory loss, constipation, headaches, diarrhea, pain in muscles, nerve pain, tingling, numbness	27% reported using cannabis for treating symptoms.<br>Patient-reported improvement of appetite (97%), muscle pain (94%), nausea (93%), anxiety (93%), nerve pain (90%), depression (86%), and paresthesia (85%).<br>47% cannabis users reported associated memory deterioration.	Unstandardized products administered by smoking.
Brunt (2014) [35]	questionnaire survey	102	herbal cannabis	different doses and patterns reported	cannabis-treated patients	n/a	• subjective effects: alertness, tranquility, confidence, dejection, dizziness, confusion/ disorientation, fatigue,	Dejection, anxiety, and appetite stimulation were found to differ among the 3 strains of cannabis.	High bias; patient-reported effects only.

							anxiety, irritability, appetite, creative stimulation, and sociability<br>• the therapeutic satisfaction of the pharmaceutical cannabis defined as the frequency of reported therapeutic effects and fulfillment of these effects in a 4-point scale<br>• comparison between THC-rich and CBD-rich strains	Patients reported therapeutic satisfaction mainly pain alleviation.	
Blaas (2008) [36]	case study	2	dronabinol	5—12,5 mg	depression, stress, burnout syndrome	1 and 6 years	• observation of depression, stress, burnout syndrome alone or in combination with other antidepressants	Dronabinol has an anti-depressive potential.	High bias.
Page (2006) [38]	qualitative study (one-to-one semistructured interviews)	14	illicitly supplied cannabis	n/a	multiple sclerosis	n/a	• patterns of use<br>• legal or social concerns<br>• perceived effects	Consumption patterns ranged from occasional to frequent.<br>Legal concerns expressed by most respondents were negligible. Social concerns centered on to whom use was revealed. The perceived benefits of use were in reduction of pain, spasms, tremors, nausea, numbness, sleep problems, bladder and bowel problems, and fatigue and, improved mood, ability to eat and drink, ability to write, and sexual functioning.	Small sample<br>High bias
Ware (2010) [39]	randomized, double-blind active-control equivalency crossover trial	23	herbal cannabis (THC 2.5%, 6%, and 9.4%)	75 mg single smoked inhalations using a titanium pipe	post-traumatic or postsurgical neuro-neuropathic pain	4 days	• The average daily pain intensity, measured on the 11-point numeric rating scale (NRS)	A single inhalation of 25 mg of 9.4% tetrahydrocannabinol herbal cannabis three times daily for five days reduced the intensity of pain, improved sleep and was well tolerated.	Small sample; short observation period; low THC concentration strains.
Lynskey (2004) [41]	cross-sectional survey of twin pairs	277 same-sex twin pairs discordant for cannabis dependence and 311 pairs discordant for early-onset cannabis use	marijuana	n/a	early-onset cannabis use suicidal ideation	n/a	• self-report measures of DSM-IV-defined lifetime major depressive disorder (MDD), suicidal ideation, and suicide attempt.	Odds of suicidal ideation and suicide attempt that were 2.5 to 2.9 times higher in cannabis dependent individuals than those of their co-twins.<br>Cannabis dependence was associated with	--

		(before age 17 years)						elevated risks of MDD in dizygotic but not in monozygotic twins. Early cannabis use initiation results in elevated rates of subsequent suicide attempt.	
**Anxiety**									
Christensen (2007) [46]	meta-analysis of randomised trials (RCTs) (4 double-blind, randomised controlled trials)	4,105 in 4 RCTs	rimonabant (CB1-receptor antagonist as an anti-obesity agent)	20 mg	obesity	12-24 months	• hospital anxiety and depression scale (HADS) score	Rimonabant increases the risk of depressed mood and anxiety.	--
Crippa (2009) [50]	systematic review	not specified number of studies and patients (253 patients in 15 experimental studies)	cannabis, THC, CBD, nabilone	detailed dosing per study	anxiety	n/a	• different anxiety scales (State-Trait Anxiety Inventory, VAS, Profile of Mood States)<br>• forearm blood flow, heart rate<br>• Subjective Drug Effects Questionnaire<br>• reported anxiety and panic	The precise relationship between cannabis use and anxiety was not established.	A narrative review; no quality assessment.
Fraser (2009) [52]	retrospective analysis of case series	47	nabilone	0.2—4.0 mg nightly	post-traumatic stress disorder (PTSD)	4 months to >2 years	• evaluation the effects of nabilone, an endocannabinoid receptor agonist, on treatment-resistant nightmares in patients diagnosed with PTSD	The majority of patients (72%) receiving nabilone experienced either cessation of nightmares or a significant reduction in nightmare intensity. Some patients noted subjective improvement in sleep time, the quality of sleep, and the reduction of daytime flashbacks and nightsweats.	High bias; no placebo control, the measurements limited to subjective reports to nightmare changes; small sample size.
Blessing (2015) [53]	systematical review of preclinical research and 12 RCTs	not specified	CBD	15—600 mg or 1 mg/kg orally; 16—32 mg inhaled	anxiety disorders	n/a	• anxiety and mood scales (State-Trait Anxiety Inventory – STAI, negative self-evaluation subscale – SSPS-N, visual analog mood scale – VAMS, visual analog scale – VAS)<br>• blood pressure<br>• skin conductance response (SCR)	Decrease in THC-induced increases in STAI, VAMS, VAS anxiety scores, blood pressure; reduction in SCR. Evidence from human studies supports an anxiolytic role of CBD, but is currently limited to acute dosing, with few studies in clinical populations.	A narrative review; no quality assessment.
Bergamaschi (2011) [54]	randomized double-blind placebo-controlled trial	24	CBD	600 mg	generalized social anxiety disorder (SAD)	a single dose	• simulation public speaking test (SPST)	CBD significantly reduced anxiety, cognitive impairment and discomfort in	Single dose effect only; preliminary results; male only.

							• subjective ratings on the visual analogue mood scale (VAMS)<br>• negative self-statement scale (SSPS-N)<br>• physiological measures (blood pressure, heart rate, and skin conductance)<br><br>at six different time points during the SPST	speech performance, and significantly decreased alert in anticipatory speech.	
Shannon (2019) [55]	retrospective analysis of case series	72	CBD	25-–175 mg	anxiety, poor sleep	≥ 1 month	• To determine whether CBD helps improve sleep and/or anxiety in a clinical population.	Anxiety scores decreased within the first month in 79.2% of patients and remained decreased during the study duration. Sleep scores improved within the first month in 66.7% of patients but fluctuated over time.	High bias; no comparison group
Crippa (2011) [56]	randomized double-blind repeated measures within-subject cross-over trial	10	CBD	400 mg	generalized social anxiety disorder (SAD)	2 sessions	• functional neuroimaging	CBD reduced anxiety in SAD through activity in limbic and paralimbic brain areas.	Small sample size; single dose effect only; preliminary results.
Zuardi (2017) [57]	randomized placebo-controlled and active-control trial	80	clonazepam<br><br>CBD	1 mg<br><br>100, 300, and 900 mg	anxiety	5 sessions	• the anxiolytic effect of cannabidiol (CBD)<br>• visual analog mood scale (VAMS)<br>• test of public speaking in a real situation (TPSRS)	Acute administration of CBD induced anxiolytic effects with a dose-dependent inverted U-shaped curve in healthy subjects.	Small sample size; no plasma levels of CBD and clonazepam.
Zvolensky (2008) [58]	observational study	1,709	cannabis		abuse, dependence and panic panic disorder in adolescents	imprecise (years)	• cannabis use, abuse, and dependence	Cannabis use and dependence were significantly prospectively associated with an increased odds for the development of panic attacks and panic disorder.<br>Cannabis was not incrementally associated with the development of panic after controlling for daily cigarette smoking.	High bias.
**Sleep disorders**									
Koethe (2009) [60]	pharmacokinetics study	20	--	--	sleep deprivation	2 sessions (second after 12 month)	• serum and cerebrospinal fluid (CSF) concentration of , the endogenous cannabinoid anandamide and its structural analog oleoylethanolamide	Oleoylethanolamide release may represent an endogenous neuroprotective signal during sleep deprivation.	Experimental research.

							before and after a night of sleep deprivation		
Hanlon (2016) [61]	randomized crossover study	14	--	--	sleep disorders	4 days	• normal (8.5 h) versus restricted sleep (4.5 h) in healthy young adults<br>• 24-h profiles of circulating concentrations of 2-AG and its structural analog 2-oleoylglycerol (2-OG)<br>• assessment hunger, appetite, and food intake under controlled conditions	Activation of the ECS may be involved in excessive food intake in a state of sleep debt and contribute to the increased risk of obesity associated with insufficient sleep.	Small sample size; no blinding procedure detailed;<br>short duration of sleep restriction; relatively low frequency of sampling (i.e., hourly) of endocannabinoid serum concentrations.
Hanlon (2015) [62]	laboratory study	14	--	--	healthy young nonobese adults	4 days	• 24-hour profile of 2-arachidonoylglycerol (2-AG) concentrations	Activity of the ECS is modulated by circadian rhythmicity and suggest that its impact on the regulation of food intake is suppressed during sleep and is maximal during early to midafternoon.	Small sample size; relatively low frequency (i.e., hourly) of sampling.
Garcia (2015) [63]	narrative review	not specified	nicotine, alcohol, opioids, cocaine, caffeine, cannabis	--	sleep disorders	n/a	• sleep disturbances using polysomnography<br>• total sleep time, sleep onset latency, rapid-eye movement (REM), REM latency, wake after sleep onset, and slow wave sleep	Low-dose THC is found to have a mild sedative effect manifested as a decrease in sleep onset latency and REM sleep and increase in total sleep time and slow wave sleep.<br>In high doses, THC decreases REM and slow wave sleep and increases sleep onset latency	Small number of studies; small sample sizes; only male subjects recruited.
Fiz (2011) [64]	cross-sectional survey	56	illicit cannabis (marijuana cannabis)	n/a	fibromyalgia (FM)	n/a	• patterns of cannabis use<br>• quality of life of FM<br>• symptom relief (Likert-style scale)<br>• perceived benefits of cannabis on a range of symptoms (pain, stiffness, relaxation, drowsiness, well-being) using 100-mm VAS scales (VAS)<br>• Fibromyalgia Impact Questionnaire (FIQ),<br>• Pittsburgh Sleep Quality Index (PSQI)<br>• Short Form 36 Health Survey (SF-36)	Statistically significant reduction of pain and stiffness, enhancement of relaxation, and an increase in somnolence and feeling of well being.<br>The mental health component summary score of the SF-36 was significantly higher in cannabis users than in non-users.<br>The use of cannabis was associated with beneficial effects on some FM symptoms.	High bias at the recruitment phase; small sample size.
Berlach (2006) [66]	retrospective analysis of case series	20	nabilone	n/a	chronic noncancer pain	4 years (average of 1.5 years)	• pain intensity in numerical rating scale (NRS)	15 patients reported subjective overall improvement and 9	High bias; small sample size.

							• changes in pain (e.g., night-time pain, pain exacerbations)	reported reduced pain intensity; beneficial effects on sleep and nausea.	
Russo (2007) [67]	narrative review of phase I-III studies	2000	THC, CBD, and THC:CBD 1:1 combination (Sativex®)	various doses detailed in each study	pain and sleep in multiple sclerosis, peripheral neuropathic pain, intractable cancer pain, and rheumatoid arthritis	up to 4 years	• effects on sleep in the context of medical treatment of neuropathic pain and symptoms of multiple sclerosis	Improvement in subjective sleep parameters with an acceptable adverse event profile. No tolerance to the benefit of Sativex on pain or sleep, nor need for dosage increases.	Bias due to narrative character of the review.
Ware (2010) [68]	randomized, double-blind active-control equivalency crossover trial	29	nabilone<br><br>amitriptyline	0.5—1.0 mg<br><br>10—20 mg	sleep disorder in fibromyalgia (FM)	2 weeks	• sleep improvement (Insomnia Severity Index)<br>• restfulness and wakefulness (Leeds Sleep Evaluation Questionnaire)	Nabilone was effective in improving sleep in patients with FM and was well tolerated. Nabilone was superior to amitriptyline and marginally better on the restfulness but not on wakefulness. No effects on pain, mood, or quality of life were observed. Low-dose nabilone given once daily at bedtime may be considered as an alternative to amitriptyline.	Small sample size.
Babson (2017) [69]	systematic review	1366 (not precisely detailed) in 25 trials and case reports	cannabis, THC, CBD, THC:CBD 1:1, dronabinol	detailed per substance and route of administration	sleep disorders in post-traumatic stress disorder	varied	• sleep quality	Mixed effects:<br>• CBD may have therapeutic potential for REM sleep behavior disorder and excessive daytime sleepiness<br>• THC may decrease sleep latency but could impair sleep quality long-term<br>• synthetic cannabinoids (nabilone, dronabinol) may have short-term benefit for sleep apnea<br>• nabilone may reduce nightmares associated with post-traumatic stress disorder (PTSD) and may improve sleep among patients with chronic pain.	Small sample size of studies and case studies; low quality of evidence.

**Schizophrenia and psychosis**									
Leweke (2012) [19]	phase II double-blinded randomized parallel-group active-control non-inferiority clinical trial	39	cannabidiol<br><br>amisulpride	200 mg increased gradually and modified to target 600-800 mg/day (200 mg three to four times a day) each	acute schizophrenia	28 days	• Brief Psychiatric Rating Scale (BPRS)<br>• Positive and Negative Syndrome Scale (PANSS) were<br>• serum levels of anandamide, palmityolethanolamide, and oleoylethanolamide	Cannabidiol appeared non-inferior to amisulpride in significant clinical improvement in acutely exacerbated schizophrenic patients, but produced significantly less adverse effects (motor symptoms, weight gain, and sexual dysfunction). Cannabidiol treatment was accompanied by a significant increase in serum anandamide levels, which was significantly associated with clinical improvement.	Relatively small sample size.
Korver (2010) [70]	cross-sectional study	121	marijuana	n/a	ultra-high-risk (UHR) of psychosis patients aged 12-35 years	--	• Structured Interview for Prodromal Syndromes (SIPS)<br>• Bonn Scale for the Assessment of Basic Symptoms–Prediction List (BSABS-P)<br>• clinical measures	Cannabis-using UHR patients have more basic symptoms than non-using patients. Healthy cannabis users have more subclinical UHR and basic symptoms and more neuropsychological dysfunctions than non-cannabis users.<br>More frequent cannabis use was related to increased severity of certain UHR symptoms.	High bias.
Henquet (2010) [71]	observational study (structured time-sampling technique)	80	marijuana	n/a	frequent cannabis users (current use of at least three times per week)	6 days	• psychotic symptoms and mood disorders in patients with psychosis and healthy controls	Patients with psychosis were more sensitive to the psychosis-inducing and mood-enhancing effects of cannabis.<br>In patients, but not in controls, cannabis use predicted increased levels of hallucinatory experiences.	Patient-reported outcomes<br>High bias
Zammit (2008) [72]	systematic review of cohort studies	not detailed no. of patients in 13 studies	marijuana	n/a	cannabis users	--	• incidence of psychotic symptoms	Cannabis use was consistently associated with increased relapse and non-adherence.	Possible underestimated association due to random misclassification of data and self-reported measures.
Vaucher (2018) [73]	meta-analysis of observational studies (Mendelian randomization study)	79,845	marijuana	n/a	cannabis users	--	• incidence of schizophrenia (odds ratio)	Based on the genetic approach, use of cannabis was associated with increased risk of schizophrenia.<br>The genetic markers did not show evidence of	Limitations typical to Mendelian randomization studies.

								pleiotropic effects and accounting for tobacco exposure did not alter the association.	
Iseger (2015) [75]	systematic review	3570 in 29 studies	CBD	varied	cannabis users	--	• hair sample analysis<br>• N-acetylaspartate, total creatine<br>• proton magnetic resonance spectroscopy, functional magnetic resonance imaging<br>• psychometric tests	CBD counteracts psychotic symptoms and cognitive impairment associated with cannabis use or acute THC administration.<br>CBD may lower the risk for developing psychosis related to cannabis use.	--
Englund (2013) [76]	randomized double-blind placebo-controlled trial	48	CBD<br><br>THC	600 mg orally<br><br>1.5 mg intravenously	healthy participants	1 day	• paranoid symptoms and hippocampal-dependent memory impairment (Positive and Negative Syndrome Scale, State Social Paranoia Scale)<br>• memory impairment (Hopkins Verbal Learning Task-revised)	High-THC/low-CBD cannabis products were associated with increased risks for mental health.<br>CBD decreased THC-induced positive psychotic symptoms and prevented against THC-induced hippocampal-dependent memory impairment.	Only one dose of CBD was investigated.<br>Short observation period.
D'Souza (2005) [77]	randomized double-blind placebo-controlled trial	13	THC	0 mg, 2.5 mg, and 5 mg intravenously	stable, antipsychotic-treated schizophrenia patients	3 days	• behavioral, cognitive, motor, and endocrine effects	THC transiently increased learning and recall deficits; positive, negative, and general schizophrenia symptoms; perceptual alterations; akathisia, rigidity, and dyskinesia; deficits in vigilance; and plasma prolactin and cortisol.<br>THC was associated with transient exacerbation in core psychotic and cognitive deficits in schizophrenia.	Small sample;<br>blinding and randomization procedures not detailed.
Zuardi (2006) [79]	case series	3	CBD<br><br>olanzapine	40—1280 mg orally<br><br>10—20 mg orally	treatment-resistant schizophrenia (TRS)	40 (+15) days	• efficacy, tolerability and safety of CBD monotherapy	Mild improvement in one of the three patients during CBD monotherapy. CBD appeared well tolerated. CBD monotherapy may not be effective for TRS.	High bias.
McGuire (2018) [80]	randomized double-blind parallel-group placebo-controlled trial	90	CBD	1000 mg	schizophrenia	6 weeks	• Positive and Negative Syndrome Scale (PANSS)<br>• Brief Assessment of Cognition in Schizophrenia (BACS)<br>• Global Assessment of Functioning scale (GAF)	CBD had beneficial effects in patients with schizophrenia, was well tolerated with rates of adverse events similar to placebo.	--

							• improvement and severity scales of the Clinical Global Impressions Scale (CGI-I and CGI-S)		
Freeman (2020) [81]	randomized placebo-controlled trial	82	CBD	200—800 mg orally	cannabis use disorder	4 weeks	• reduction in urinary THC-COOH:creatinine ratio; an increase in days per week with abstinence from cannabis; or both	Cannabidiol 400 mg and 800 mg were safe and more efficacious than placebo at reducing cannabis use.	Magnitude of efficacy not assessed due to a type of study.
Boggs (2018) [82]	randomized double-blind placebo-controlled trial	36	CBD	600 mg orally	schizophrenia	6 weeks	• cognitive, symptomatic, and side effects of CBD<br>• augmentation in antipsychotic-treated patients diagnosed with chronic schizophrenia<br>• MATRICS Consensus Cognitive Battery (MCCB)<br>• Positive and Negative Syndrome Scale (PANSS)	CBD augmentation was not associated with an improvement in MCCB or PANSS scores in stable antipsychotic-treated outpatients with schizophrenia. CBD was well tolerated with no worsening of mood, suicidality, or movement side effects.	Likely too low dose (600 mg/day) to reveal beneficial effects of CBD on psychosis and possibly cognitive impairments associated with schizophrenia; small sample; blinding and randomization procedures not detailed; high bias.
Wilkinson (2014) [83]	review of selected studies	87,700 patients in 4 epidemiological studies and 7,065 patients in 6 genetic-cannabis interaction studies	marijuana	n/a	cannabis users	up to 38 years	• incidence of psychotic disorders	THC and synthetic cannabinoids induce transient positive, negative, and cognitive symptoms in healthy volunteers. Cannabinoids can induce acute psychosis that lasts beyond the period of acute intoxication but resolves within a month. Exposure to cannabis in adolescence is associated with a risk for later psychotic disorder in adulthood.	Narrative review of recent evidence.
Gage (2016) [84]	review of epidemiological studies	106,700 patients in 10 studies	marijuana	n/a	cannabis users	n/a	• incidence of psychotic disorders	Strong evidence that cannabis use can increase the risk of psychotic disorders.	Magnitude effect not assessed.
D'Addario (2017) [85]	laboratory study	131	--	--	bipolar disorder, major depressive disorder, and schizophrenia	--	• selective alterations of DNA methylation at the promoter of CNR1 (the gene coding for the type-1 cannabinoid receptor)	Findings suggest a selective dysregulation of endocannabinoid system in psychosis, and highlight the evaluation of CNR1 DNA methylation levels in peripheral blood mononuclear cells as a potential biomarker for schizophrenia.	Small sample size for schizophrenia (N=25). Schizophrenia patients showed residual/active positive symptoms, due to the recruitment setting.

**Cognitive disorders and dementia**									
Pope (2002) [90]	retrospective study	164	marijuana	n/a	current heavy cannabis users (at least 5,000 times)	28 days	• Buschke's Selective Reminding Test (BSRT; a<br>• test of memory of word lists),<br>• continuous performance tests,<br>• the Benton Visual Retention Test (BVRT; a test of visuospatial memory)<br>• vocabulary subscale of the Wechsler Adult Intelligence Scale (WAIS-R; a measure of general intellectual ability)	Heavy marijuana users showed deficits on memory of word lists on the first 7 days of a supervised abstinence period. This effect weaned by Day 28, which suggests that cannabis-associated cognitive deficits are reversible and related to recent cannabis exposure rather than irreversible and related to cumulative lifetime use.	Bias typical to retrospective studies.
Bolla (2002) [91]	observational study	22	marijuana	2—117 joints/week	heavy marijuana users	28 days	• neurocognitive deficits persist in 28-day abstinence period<br>• neurocognitive tests<br>• Drug Use Survey Questionnaire (DUSQ)<br>• Addiction Severity Index (ASI)<br>• Diagnostic Interview Schedule (DIS)	Very heavy use of marijuana is associated with persistent decrements in neurocognitive performance (memory, executive functioning, and manual dexterity) during 28 days of abstinence.<br>There was a negative association between marijuana use and executive cognitive functioning.	The Bonferroni correction was not applied for multiple comparisons; no definitive statements about causality can be made; small sample size.
Krishnan (2009) [97]	systematical review	15 patients in 1 trial—Volicer (1997) [98]	dronabinol	2.5 mg bid orally	probable Alzheimer's dementia with simple food refusal, normal blood tests or no significant laboratory abnormalities	6 weeks	• Weight gain, body mass index (BMI), triceps skin fold thickness, caloric intake<br>• Disturbed behavior (Cohen-Mansfield Agitation Inventory score)<br>• Affect (Lawton Observed Affect Scale - Past)<br>• Plasma albumin and lymphocyte count	There was no evidence that cannabinoids are effective in the improvement of disturbed behavior in dementia or in the treatment of other symptoms of dementia.	Only one study met the inclusion criteria.
van den Elsen (2015) [99]	randomized double-blind placebo-controlled study	50	THC 1.5 mg tid		dementia-related neuropsychiatric symptoms (NPS)	3 weeks	• Neuropsychiatric Inventory (NPI)	THC does not significantly reduce dementia-related NPS, though it is well-tolerated.	--
**Opioid withdrawal symptoms and drug substitution**									
Scavone (2013) [102]	retrospective study	91	methadone, marijuana	n/a	opiate-dependent cannabis users on methadone maintenance treatment (MMT)	> 9 months	• patterns of cannabis use prior to and during MMT<br>• cannabis-related effects on MMT	Patients who used marijuana before MMT needed less methadone. Rates of cannabis use were high during methadone induction	Descriptive nature of analysis Patient-reported data on substance use history prior to MMT.

							• clinical opiate withdrawal scale (COWS)	and decreased following dose stabilization.<br>The cannabis use during methadone therapy resulted in less expressed withdrawal symptoms (COWS).	
Hurd (2019) [103]	randomized double-blind placebo-controlled trial	42	CBD	400 or 800 mg for 3 consecutive days	1 heroin use disorder	7 days	• reduction in cue-induced craving and anxiety	CBD significantly reduced craving and anxiety induced by the presentation of salient drug cues compared with neutral cues with significant protracted effects on these measures 7 days after the 3-day CBD exposure.<br>CBD reduced the drug cue-induced physiological measures of heart rate and salivary cortisol levels.<br>There were no significant effects on cognition, and there were no serious adverse effects.	Small sample size.
Lucas (2013) [106]	questionnaire survey	404	herbal cannabis	n/a	herbal cannabis patients	--	• subjective impact of medical cannabis on the use of both licit and illicit substances via self-report<br>• substitution effect, in which the use of one product or substance is influenced by the use or availability of another	Over 75% of respondents substituted cannabis for at least one other substance: over 41% for alcohol; 36.1% for illicit substances; 67.8% for prescription drugs.<br>The reasons for cannabis as a substitute were: "less withdrawal" (67.7%), "fewer side-effects" (60.4%), and "better symptom management".	Patient-reported subjective assessment.
Epstein (2015) [109]	retrospective study	116	marijuana	n/a	outpatient heroin and cocaine users on the methadone dose taper	10 weeks	• weekly urine screens for cannabinoids<br>• every-two-week assessments of opioid-withdrawal symptoms	Opioid-withdrawal scores did not differ overall between users and nonusers of cannabis.<br>The evidence does not support smoked cannabis as a reducer of opioid-withdrawal symptoms in the context of a methadone dose taper.	Relatively small sample size; urine drug tests were qualitative, precluding biochemical assessment of the amounts of drugs used; assessment of opioid-withdrawal symptoms once every two weeks and only over a time frame of the past two days.
Jicha (2015) [110]	randomized double blind	12	oxycodone<br><br>dronabinol	30 and 60 mg	adult volunteers physically dependent on	5 weeks	• heart rate, blood pressure, respiratory	Dronabinol 20-30 mg produced increases in heart rate up, and 40	--

	placebo-controlled trial			5, 10, 20, and 30 mg [decreased from 40 mg])	short-acting opioids (oxycodone 30 mg qid)	(7 sessions of 21-hour opioid withdrawal)	outcomes, and pupil diameter<br>• safety assessment	mg—sustained sinus tachycardia accompanied by anxiety and panic. Dronabinol 5 and 10 mg produced placebo-like effects. High dose dronabinol may have limited utility as stand-alone agent to treat acute opioid withdrawal.	
Pacheco-Colón (2018) [111]	systematic review	22 studies	marijuana	n/a	cannabis users	--	• decrease in motivation among cannabis users<br>• causal relationship between cannabis use and motivation	Although cross-sectional evidence of a cannabis-specific effect on motivation is equivocal, there is partial support from longitudinal studies for a causal link between cannabis use and reduced motivation.	--

bid—twice a day; CBD—cannabidiol; ECS—endocannabinoid system; n/a—not available or not applicable; qid—four times a day; QoL—Quality of life; RCT—randomized controlled trial; THC—tetrahydrocannabinol; tid—three times a day

Note: marijuana—illicit unstandardized (‘street’) cannabis; herbal cannabis—‘medical’ standardized cannabis
